# New perspective: Symbiotic pattern and assembly mechanism of *Cantharellus cibarius*-associated bacteria

**DOI:** 10.3389/fmicb.2023.1074468

**Published:** 2023-02-16

**Authors:** Wei Ge, Yulian Ren, Chunbo Dong, Qiuyu Shao, Yanmin Bai, Zhaoying He, Ting Yao, Yanwei Zhang, Guosheng Zhu, Sunil Kumar Deshmukh, Yanfeng Han

**Affiliations:** ^1^Institute of Fungus Resources, Department of Ecology/Key Laboratory of Plant Resource Conservation and Germplasm Innovation in Mountainous Region (Ministry of Education), College of Life Sciences/Institute of Agro-Bioengineering, Guizhou University, Guiyang, Guizhou, China; ^2^Analysis and Test Center, Huangshan University, Huangshan, China; ^3^School of Biological Sciences, Guizhou Education University, Guiyang, Guizhou, China; ^4^Guizhou Key Laboratory of Edible Fungi Breeding, Institute of Crop Germplasm Resources, Guizhou Academy of Agricultural Sciences, Guiyang, Guizhou, China; ^5^TERI-Deakin Nano Biotechnology Centre, The Energy and Resources Institute, New Delhi, India

**Keywords:** *Cantharellus cibarius*, mycosphere, diversity, community assembly, microbial interaction, volatile organic compounds

## Abstract

*Cantharellus cibarius*, an ectomycorrhizal fungus belonging to the Basidiomycetes, has significant medicinal and edible value, economic importance, and ecological benefits. However, *C. cibarius* remains incapable of artificial cultivation, which is thought to be due to the presence of bacteria. Therefore, much research has focused on the relationship between *C. cibarius* and bacteria, but rare bacteria are frequently overlooked, and symbiotic pattern and assembly mechanism of the bacterial community associated with *C. cibarius* remain unknown. In this study, the assembly mechanism and driving factors of both abundant and rare bacterial communities of *C. cibarius* were revealed by the null model. The symbiotic pattern of the bacterial community was examined using a co-occurrence network. Metabolic functions and phenotypes of the abundant and rare bacteria were compared using METAGENassist2, and the impacts of abiotic variables on the diversity of abundant and rare bacteria were examined using partial least squares path modeling. In the fruiting body and mycosphere of *C. cibarius*, there was a higher proportion of specialist bacteria compared with generalist bacteria. Dispersal limitation dominated the assembly of abundant and rare bacterial communities in the fruiting body and mycosphere. However, pH, 1-octen-3-ol, and total phosphorus of the fruiting body were the main driving factors of bacterial community assembly in the fruiting body, while available nitrogen and total phosphorus of the soil affected the assembly process of the bacterial community in the mycosphere. Furthermore, bacterial co-occurrence patterns in the mycosphere may be more complex compared with those in the fruiting body. Unlike the specific potential functions of abundant bacteria, rare bacteria may provide supplementary or unique metabolic pathways (such as sulfite oxidizer and sulfur reducer) to enhance the ecological function of *C. cibarius*. Notably, while volatile organic compounds can reduce mycosphere bacterial diversity, they can increase fruiting body bacterial diversity. Findings from this study further, our understanding of *C. cibarius*-associated microbial ecology.

## 1. Introduction

Mycorrhizal is one of the most common symbiotic forms in terrestrial ecosystems. More than 80% of plants in the world form mycorrhizal relationships by interacting with various types of fungi, such as ectomycorrhiza (Chauhan et al., 2022). The fungi that form ectomycorrhiza in symbiosis with plants are known as ectomycorrhizal fungi (EcMF) and include most Basidiomycota and Asomycota and some Zygomycota. These fungi predominantly associate with the roots of some plants in Pinaeceae, Cupressaceae, and Salicaceae to form EcMF with unique structures such as mantle, Harley reticulum, and extramatrical mycelia (Chot and Reddy, 2022). The development of the ecological niche of other soil microorganisms (such as bacteria) is thought to have been significantly influenced by the origin of coevolution between mycorrhizal fungi and land plants, leading to a number of antagonistic and reciprocal strategies (Boer et al., 2005). Mycorrhizal fungi have become a research hotspot in microbial ecology owing to the rapid development of various disciplines and microbial technologies, which have facilitated the gradual realization of the significance of fungus-related microbiota in fruiting body formation and development (Deveau et al., 2016; Pent et al., 2017). For instance, extensive research has been conducted on the microbiota of Ascomycetes (truffles) (Antony-Babu et al., 2014; Splivallo et al., 2015; Liu et al., 2021), while less is known about the microbiota of traditionally consumed Basidiomycetes. A group of EcMF known as *Cantharellus cibarius* is extensively distributed and has a delectable flavor and a high economic value. The presence of bacteria and other foreign microorganisms in the spores and tissues of the fruiting body, which complicates the intricate symbiotic relationship between the fruiting body and host tree, is thought to be the primary reason why artificial cultivation of *C. cibarius* is challenging (Kozarski et al., 2015). Consequently, rapidly developing high-throughput sequencing technologies have been employed to supplement the understanding of the microbial species associated with *C. cibarius* (Pent et al., 2017, 2020; Gohar et al., 2020; Ge et al., 2021). For instance, Gohar et al. (2020) found that the potential functional abundance of bacteria connected to *C. cibarius* varied with tissue and developmental phases. Ge et al. (2021) discovered differences in bacterial community composition and potential functional differentiation trends between fruiting bodies and rhizomorphs of *C. cibarius*. According to Pent et al. (2017, 2020), the main predominant factors influencing the bacterial diversity and community composition of *C. cibarius* were soil pH, host identity, and chemical composition of fruiting bodies. Saidi et al. (2016) isolated six strains of bacteria that could produce aroma from the fruiting body of *C. cibarius*, but it was unclear whether these bacteria were involved in the aroma synthesis of fungi. However, a similar situation has been demonstrated in Ascomycota such as truffles (Splivallo et al., 2015; Orban et al., 2023). Typically, the volatile organic compounds (VOCs) in the odor components of mushrooms are the primary factors affecting odor characteristics (Zhu et al., 2022). Such VOCs are low-molecular-weight substances (up to 500 Da) with high vapor pressure and a low boiling point. These properties are conducive to VOCs being volatilized and diffused in soil and air, even over long distances (Tyc et al., 2017; Orban et al., 2023). Consequently, VOCs are particularly suitable as signaling substances to assist mushrooms in being easily detected by animals or humans and completing their spore propagation strategy (Vahdatzadeh et al., 2019; Ge et al., 2021). VOCs also frequently mediate interactions between soil microorganisms, but there is limited information on whether VOCs also affect *C. cibarius*-associated microorganisms.

Many low-abundance taxa and a small number of dominating taxa make up soil microbial communities, according to an increasing number of studies (Zheng et al., 2021). Rare taxa frequently exhibit great diversity and functional redundancy as a member of the microbial “seed bank,” and they play a significant ecological role in preserving species diversity and ecosystem function (Lynch and Neufeld, 2015; Jousset et al., 2017; Chen et al., 2020). They frequently exhibit considerable interactions with abundant taxa, forming an intricate ecological network (Jousset et al., 2017). Network analyses of symbiotic patterns provide new insights into such complex ecological networks, helping to reveal niche spaces or symbiotics shared by community members (Barberán et al., 2012; Faust and Raes, 2012). Therefore, exploring symbiotic patterns among microorganisms is beneficial to identify potential biological interactions, habitat affinity, or common physiology that can guide more targeted research or experimental settings (Barberán et al., 2012). Uncertainty about the interaction and symbiotic patterns of bacterial communities’ results from the fact that many datasets of bacterial taxa related to *C. cibarius* do not directly show evidence for species interactions. Instead, they tend to focus on abundant taxa while ignore or delete rare taxa.

In recent years, researchers have found that microorganisms most likely participate in the whole process of EcMF from hyphal growth and ectomycorrhizal formation to fruiting body development (Antony-Babu et al., 2014; Li et al., 2018; Baragatti et al., 2019). For example, archaea, fungi, viruses, and protozoa can be symbiotic partners of mycorrhizal, hyphae, and fruiting bodies (Bomberg et al., 2003; Antony-Babu et al., 2014; Marupakula et al., 2017). The factors driving the assembly and symbiosis of these microorganisms may be caused by differences in host exudate composition or soil heterogeneity (host modification soil creation/soil intrinsic property); however, the factors that dominate the distribution and symbiotic patterns of microbial communities in the host and its unique habitat remain have yet to be elucidated (Ning et al., 2019; Jiao et al., 2020; Upadhyay et al., 2022). Soil contains rich and diverse bacterial communities, which provide a species pool for the microbiota of soil organisms (Antony-Babu et al., 2014; Deveau et al., 2016; Gohar et al., 2020). Thus, environmental forces that shape microbial community composition in the soil may indirectly contribute to the construction of microbial communities in fungal hyphae and fruiting bodies (Warmink et al., 2009; Antony-Babu et al., 2014). Studying the mechanisms underlying distribution patterns and species coexistence of microbial communities, however, remains a key issue in microbial ecology because the factors that lead to the formation of these microbial communities are poorly understood (Ning et al., 2019; Jiao and Lu, 2020; Dong et al., 2022). In recent years, there has been increasing use of community assembly to describe the distribution patterns of microbial communities, which are influenced by deterministic processes caused by microbial characteristics (e.g., phenotypic characteristics), interactions, and environmental conditions, and stochastic processes caused by microbial birth, death, colonization, extinction, and species formation (Chase and Myers, 2011; Chase, 2014; Zhou and Ning, 2017; Gao et al., 2020). However, the assembly process of unique bacterial communities in *C. cibarius* is still unknown.

This study used 12 fruiting bodies of *C. cibarius* and their corresponding mycosphere soil samples from three plots in Guiyang, Guizhou Province, China. The main objectives were (1) to explore the co-occurrence patterns and metabolic functions of abundant and rare bacteria in the fruiting bodies and mycosphere of *C. cibarius*; (2) to reveal the assembly mechanism of bacterial taxa in the fruiting body and mycosphere of *C. cibarius*; (3) to analyze the regulatory effects of various environmental factors on the diversity of abundant and rare bacteria in fruiting bodies and mycosphere. We hypothesized that: (1) the symbiotic pattern of the bacterial community in the mycosphere is more complex than the fruiting body, and the rare bacteria have some unique or complementary potential functions. (2) The assembly of abundant and rare bacterial communities in fruiting bodies and mycosphere was dominated by stochastic processes, but also influenced by different environmental factors. (3) The diversity of abundant and rare bacteria in fruiting bodies and mycosphere may be regulated differently by the same environmental factors.

## 2. Materials and methods

### 2.1. Sample collection and processing

**Figure 1:**
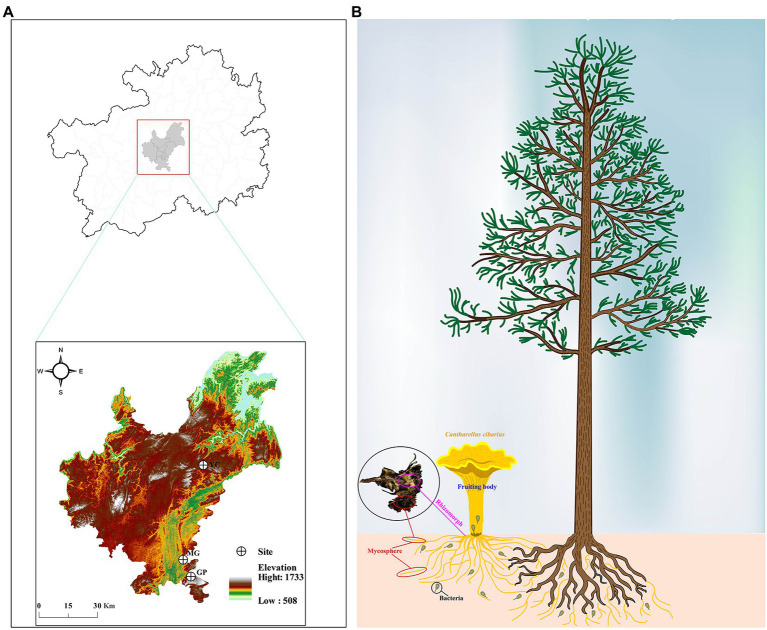
Information of the sampling site and part. **(A)** Geographic information of three sampling sites. **(B)** The habitat and sampling part of *Cantharellus cibarius*. GP, Gaopo Township; MG, Mengguan Township; YC, Yangchang Town.

The fruiting bodies and mycosphere samples were collected in August 2021 from the forest-soil region of three plots in Guiyang, Guizhou, China (see Figures 1A,B for detailed geographic information). Each fruiting body by using the measurement database^1^ is evaluated in terms of its color and morphological characteristics (size, shape, and texture). Four fruiting body samples with the same developmental period (middle-aged) and their corresponding mycosphere soil (10 cm in diameter and 5 cm in depth) were collected from each plot. The spacing between samples was more than 50 m to ensure that samples came from different host plant roots. Samples were rapidly transported to the laboratory at 4°C for processing (Warmink and van Elsas, 2008; Oh et al., 2016). The fungal material was processed in the laboratory according to the methods of Ge et al. (2021). Briefly, the fruiting bodies were separated from the soil and the soil (~2 g) adhering to the rhizomorph (hyphae) was carefully removed. All samples were processed and placed in sterile centrifuge tubes for total DNA extraction. The remaining fruiting body tissue (cap and stipe) was dried in a freeze dryer and then ground to powder (<2 mm) for nutrient analysis and volatile composition determination (Kranabetter et al., 2019). The remaining portion of the mycosphere was air-dried and sieved on a 2-mm grid before being used for physicochemical analysis.

### 2.2. DNA extraction, PCR amplification, Illumina MiSeq sequencing, and processing of sequencing data

Total genomic DNA was extracted from fruiting bodies and mycosphere samples of *C. cibarius* using the E.Z.N.A. ® soil DNA Kit (Omega Bio-Tek, Norcross, GA, USA) according to the manufacturer’s protocols. DNA concentration and purity were determined by NanoDrop 2000 UV-cis spectrophotometer (Thermo Scientific, Wilmington, USA). Variable V3-V4 regions of the 16S rRNA gene were amplified using the bacterial primers 338F (5′-ACTCCTACGGGAGGCAGCAG-3′) and 806R (5′-GGACTACHVGGGTWTCTAAT-3′) in the GeneAmp 9,700 PCR system (ABI, USA). The conditions and mixtures for PCR amplification were referenced in our previous method (Ge et al., 2021). Sequencing was performed on Illumina’s Miseq PE300 platform (Shanghai Magi Biomedical Technology Co., LTD.).

Raw data files were quality-filtered by fastp^2^ (Chen et al., 2018) and merged by FLASH^3^ with the following criteria (Magoc and Salzberg, 2011): (1) reads were truncated at any site receiving an average quality score < 20 over a 50-bp sliding window; (2) according to the overlap relation between PE reads, pairs of reads were merged into a sequence with a minimum overlap length of 10 bp; (3) the maximum mismatch ratio allowed in the overlap region of the merged sequence was 0.2, and the non-conforming sequence was screened; (4) according to barcodes and primers at the beginning and end of the sequence, the samples were distinguished, and the sequence direction was adjusted. The allowable mismatch number of barcodes was 0, and the maximum primer mismatch number was 2. Sequences with ≥97% similarity were assigned to the same operational taxonomic units (OTUs), the chimeras were filtered by using UPARSE^4^ (Stackebrandt and Goebel, 1994; Edgar, 2013) and the OTUs represented by less than two sequences were removed to avoid possible biases (Frøslev et al., 2017; Xiong et al., 2021). OTUs were classified and annotated by the RDP classifier (http://rdp.cme.msu.edu/version 2.2), then compared with the SILVA 16S rRNA database (v138), setting the comparison threshold at 70% (Wang et al., 2007). The raw data was uploaded to the NCBI database (BioProject ID: PRJNA871198; BioSample: SAMN30410437).

### 2.3. Analysis of climate, chemical composition of fruiting body, and soil physicochemical properties

Mean annual temperature (MAT), mean annual rainfall (MAR), relative humidity (RH), and sunshine duration (SD) in 2021 were obtained from meteorological data provided by the China Meteorological Data Service Center^5^ and the mean values were calculated (Supplementary Table S1). According to the method of “Physical and Chemical Analysis of Soil Properties” (Ren et al., 2019), the soil physicochemical properties and the chemical composition of the fruiting body were determined. The total nitrogen in the soil (TN) and fruiting bodies (FTN) was measured with the semi-micro-Kelvin method (LY/T1228–1999). The total phosphorus in soil (TP) and fruiting bodies (FTP) was measured by the acid digestion Mo-Sb colorimetric method (LY/T1232-1999). The total potassium of soil (TK) and fruiting bodies (FTK) was measured by the base digestion-flame photometric method (LY/T1234-1999). Soil pH was measured with the potentiometric method (LY/T1239-1999). Soil organic matter (SOC) was measured with the potassium dichromate volumetric method (LY/T1237-1999). Soil available nitrogen (AN) was measured with the alkali N-proliferation (LY/T1229-1999). Soil available phosphorus (AP) was measured with the molybdenum-blue colorimetric method (GB12297-1990). Soil available potassium (AK) was measured by the flame photometric method (LY/T1236-1999). The data obtained for all indicators were shown in Supplementary Table S1.

### 2.4. Determination of volatile organic compounds in the fruiting bodies

According to Huang et al. (2018), there is a large amount of 1-octen-3-ol and unique β-ionone and dihydro-β-ionone in the VOCs of the fruiting body of *C. cibarius*. Therefore, these three VOCs were selected for analysis in this study. After the dried fruiting bodies were crushed, 0.5059 g was weighed into a 100-mL headspace injection bottle and 20 ml saturated sodium chloride solution was added. The extraction head was desorbed for 5 min (50/30 μm PDMS extraction head), inserted into the extraction bottle, and the headspace of 70°C water bath was extracted for 60 min at 1000 R/min speed. It was quickly inserted into the injection port of the gas chromatograph, desorbed at 250°C for 5 min, and simultaneously detected by gas chromatography–mass spectrometry (GC–MS). The chromatographic column was an Agilent HP-5MS elastic capillary column (30 m × 250 μm × 0.25 μm), and the column program was 40°C for 2 min; 5°C/min to 85°C for 2 min; 2°C/min to 110°C for 1 min; 3°C/min to 128°C for 1 min; 2°C/min to 145°C for 2 min; 5°C/min to 230°C for 8 min (total run time 69 min). The flow rate was 1.0 ml/min. The linear relationships were 0.1, 0.25, 0.5, 1.0, and 2.0 μl of standard solution, and with the injection volume as the abscissa (X) and the peak area (Y) as the ordinate, standard curves were generated and the contents of various VOCs were calculated, respectively. Data of all indexes are shown in Supplementary Table S1.

### 2.5. Habitat specialization

To help explain the pattern of beta diversity, we estimated Levins’ niche breadth (*B*) index for the abundant and rare subcommunities (Levins, 1968):


$$B_{j}=\frac{1}{\Sigma_{i=1}^{N}\;P_{ij}^{2}}$$

where *B_j_* represents the niche breadth of OUT*j*; N is the total number of communities; *P_ij_* is the proportion of OTU *j* in the community *i* (Jiao et al., 2020). The *B_j_* of the fruiting body and mycosphere samples were calculated, respectively, (*N* = 12 for both). The *B* index considered habitat utilization based on species abundance and evenness at the metacommunity scale. Higher *B*-values indicate a wider niche breadth for species existing in more habitats; lower *B*-values indicate a narrower niche breadth for species in fewer habitats. We calculated the average niche breadth index (*B*_m_) for all taxa of a bacterial community to indicate the habitat utilization and divided each sub-community with an average *B*_m_ value based on 100 Bootstraps. According to the emergence of OTUs and using the permutation algorithms as implemented in EcolUtils (Salazar, 2015), OTUs were further classified as the generalist, specialist and neutral taxa (Zhang et al., 2018a).

### 2.6. Data analysis

To assess the role of abundant and rare bacteria in the fruiting body and mycosphere in community structure, the relative abundance of OTU ≥0.01% in samples was defined as abundant taxa (Dong et al., 2021), and the relative abundance of OTU <0.01% was defined as rare taxa (Jiao et al., 2017). The co-occurrence patterns of bacteria in the fruiting body and mycosphere were determined by network analysis. In order to increase statistical confidence, OTUs that were present in at least two-thirds (8 samples) of the samples were included in the network analysis, as stated by Du et al. (2020). In order to build the network, Spearman rank correlation coefficients between OTUs were determined using the “Hmisc” package (Harrell and Dupont, 2017). Only strongly correlated (|*r*| > 0.8) and statistically significant (*p* < 0.01) OTUs are present in the network (Hu et al., 2017; Du et al., 2020). The screened OTUs were visualized by Gephi (Version 0.9.2). The network topology parameters (including average clustering coefficient, average path distance, average degree, graph density, and modularity) are calculated in Gephi, version 0.9.2. In order to define the potential contribution of the deterministic and stochastic process to the assembly of microbial communities in the fruiting body and mycosphere, we used the null model in R (999 simulations) (Stegen et al., 2013) to calculate the β-nearest taxon index (|βNTI|), |βNTI| ≥ 2 and |βNTI| < 2 represent the deterministic and stochastic process of the microbial community, respectively (Jiao et al., 2020). The relative influence of dispersal limitations was quantified as the fraction of pairwise comparisons with |βNTI| < 2 and RC_Bray_ > 0.95. The fraction of all pairwise comparisons with |βNTI| < 2 and |RC_Bray_| < 0.95 was used to estimate the influence of the “undominant” (Zhou and Ning, 2017). To assess the major factors that affected the assembly processes for abundant and rare taxa, a Mantel test based on Spearman’s correlation coefficients was conducted to compare the βNTI values with the Euclidean distance matrices for each of the variables (Vass et al., 2020; Zhao et al., 2022).

In order to compare the differences in the alpha diversity of the abundant and rare bacteria in the fruiting bodies and mycosphere, we calculated the alpha diversity indexes (Shannon, Richness) and Community Richness (ACE, Chao1) by Mothur software (Supplementary Table S2). ANOVA and T-test were used to analyze the differences in alpha diversity (Schloss et al., 2011). In Statistical Analysis of Metagenomic Profiles (STAMP), ANOVA was used to calculate the beta diversity of abundant and rare bacteria in fruiting bodies and mycosphere. It will be presented in the form of PCoA (Parks et al., 2014). The “Metacoder” function in the R package was used to generate a heat tree describing the taxon abundance of the top 50 abundant and rare bacterial operational taxonomic units (OTUs) in the fruiting body and mycosphere (Foster et al., 2017). To further understand the complex relationship between climate factors (RH, MAR, MAT, and SD), physicochemical properties (total nitrogen, total phosphorus, and total potassium) of the fruiting body, VOCs (1-octen-3-ol, dihydro-β-ionone, and β-ionone), and alpha diversity (Richness, Shannon, Pielou and Chao1 indexes) of abundant and rare bacteria in the fruiting body, a partial least squares model (PLS-PM) was constructed in SmartPLS3 (Ringle et al., 2015). Similarly, a PLS-PM was also used to further explain the influence process of climate factors, soil physicochemical properties (pH, organic matter, total nitrogen, available nitrogen, total phosphorus, available phosphorus, total potassium, available potassium) and VOCs on the alpha diversity of abundant and rare bacteria in the mycosphere (Richness, Shannon, Pielou and Chao1 indexes), respectively.

The metabolic functions, energy sources and biological relationships of abundant and rare bacteria in fruiting bodies and mycospheres soils were annotated based on METAGENassist2 (Arndt et al., 2012). The phenotype information of 11,000 organisms and the full sequences of about 1,800 organisms are collected in METAGENAssisted. In addition, phenotypic information contains approximately 20 categories for each microorganism, such as metabolism, habitat, energy, oxygen demand, and preferred temperature range (Dong et al., 2022).

## 3. Results

### 3.1. Analysis of community composition and diversity of abundant and rare bacteria in the fruiting body and mycosphere

**Figure 2:**
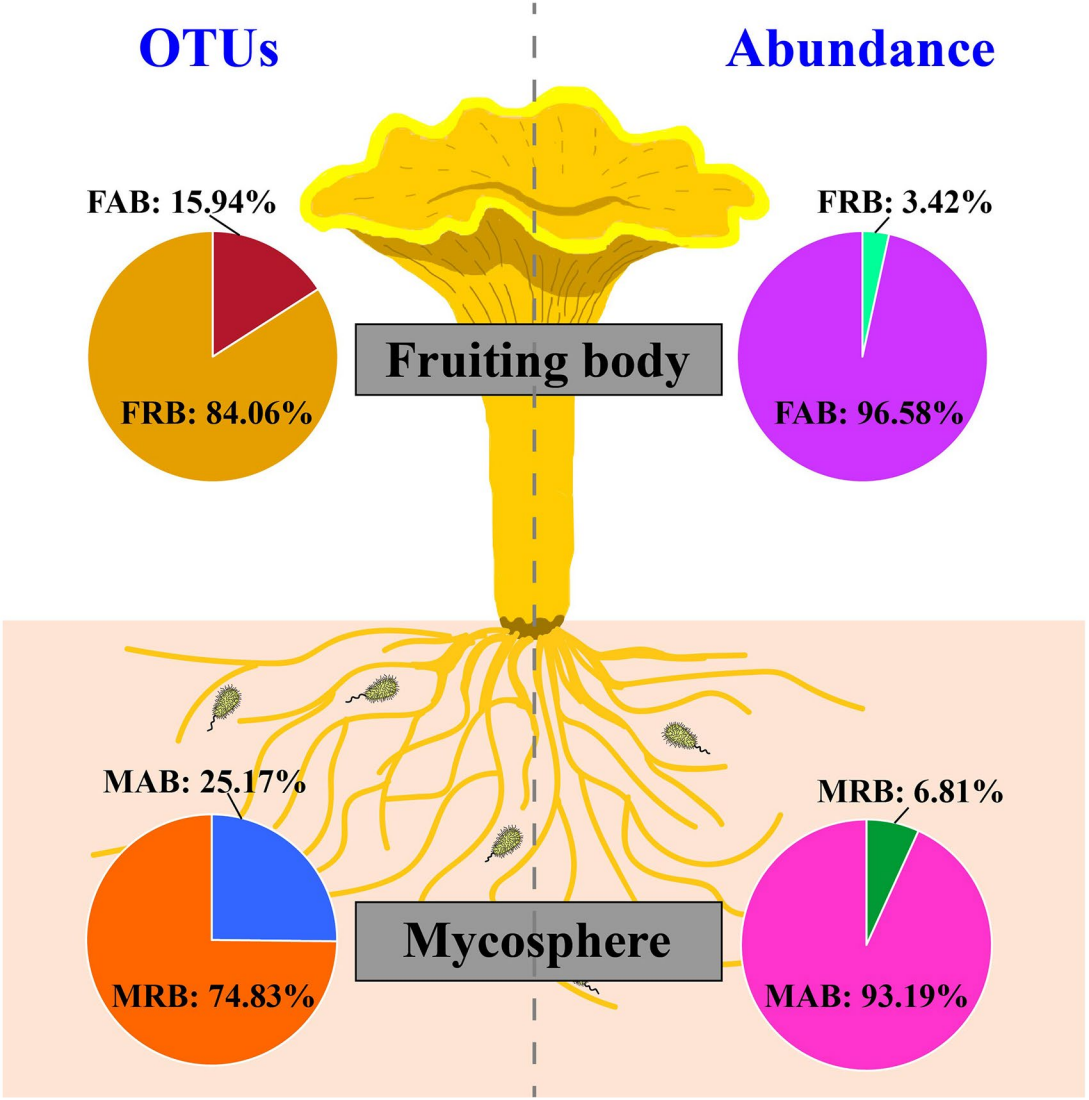
OTU and the relative abundance ratio of abundant and rare bacteria in the fruiting body and mycosphere. Abbreviation: FAB, abundant bacteria of the fruiting body; FRB, rare bacteria of the fruiting body; MAB, abundant bacteria of the mycosphere; MRB, rare bacteria of the mycosphere.

After strict quality filtering, resulting sequences were gathered into OTUs with similarity ≥97%. In all, 3,456 OTUs and 2,165 OTUs were detected in the mycosphere and fruiting body, respectively. Although a large percentage of OTUs in fruiting bodies were recognized as rare taxa (average value =84.06%), they only made up 3.42% of the average relative abundance of each sample (Figure 2). In the mycosphere, a large percentage of OTUs were classified as rare taxa (average value =74.83%), however they only accounted for 6.81% of each sample’s average relative abundance (Figure 2). The results showed that the abundant and rare bacteria had obvious distribution patterns in both fruiting bodies and mycosphere.

**Figure 3:**
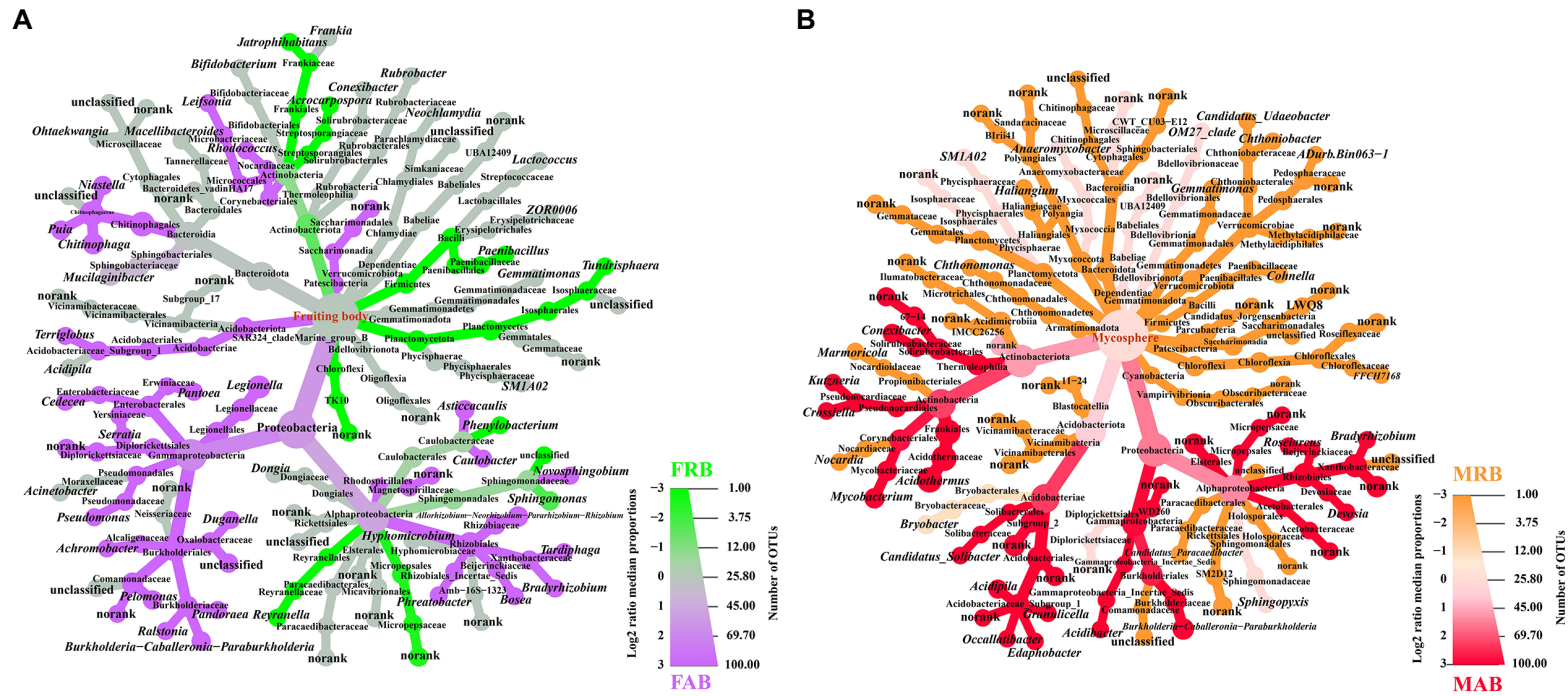
Heat tree of community classification and composition of the fruiting bodies and mycosphere. **(A)** Top 50 OTUs of abundant and rare bacteria of the fruiting body. **(B)** Top 50 OTUs of abundant and rare bacteria of the mycosphere. The size of the node is proportional to the number of OTUs within each taxon. Each figure shows the specific name of each classification level; the color scale and ruler on the right represent the log2 value of the median proportion and the number of OTUs, respectively; the colored picture shows the significantly different species compared between the two groups. Only the significantly different species will be colored, where purple represents the abundant bacteria of the fruiting body, green represents the rare bacteria of the fruiting body, red represents the abundant bacteria of the mycosphere, and orange represents the rare bacteria of the mycosphere. FAB, abundant bacteria of the fruiting body; FRB, rare bacteria of the fruiting body; MAB, abundant bacteria of the mycosphere; MRB, rare bacteria of the mycosphere.

To visualize the dominant taxa of abundant and rare bacteria in the fruiting body and mycosphere of *C. cibarius*, a heat tree was used to characterize the taxonomic information of the top 50 OTUs of abundant and rare bacteria in each sample. The abundant bacteria of the fruiting body (FAB) were mainly Proteobacteria, Acidobacteriota, and Patescibacteria at the phylum level, and *Allorhizobium-Neorhizobium-Pararhizobium-Rhizobium*, *Burkholderia-caballeronia-paraburkholderia*, *Serratia* and *Chitinophaga* were the dominant genera. The rare bacteria of the fruiting body (FRB) predominantly belonged to the phyla Planctomycetota, Firmicutes, and Actinobacteriota, and *Jatrophihabitans*, *Sphingomonas*, *Paenibacillus*, and *Reyranella* were the dominant genera (Figure 3A).

The abundant bacteria of the mycosphere (MAB)were mainly Proteobacteria, Actinobacteriota, and Acidobacteriota at the phylum level, and the dominant genera of abundant bacteria included *Acidipila*, *Bradyrhizobium*, *Devosia*, and *Crossiella*. In contrast, the rare bacteria of the mycosphere (MRB) predominantly belonged to the phyla Verrucomicrobiota, Myxococcota, Bacteroidota, and Chloroflexi, and the dominant genera of rare bacteria included *Chthoniobacter*, *Gemmatimonas*, *Cohnella*, and *Crossiella* (Figure 3B).

**Figure 4:**
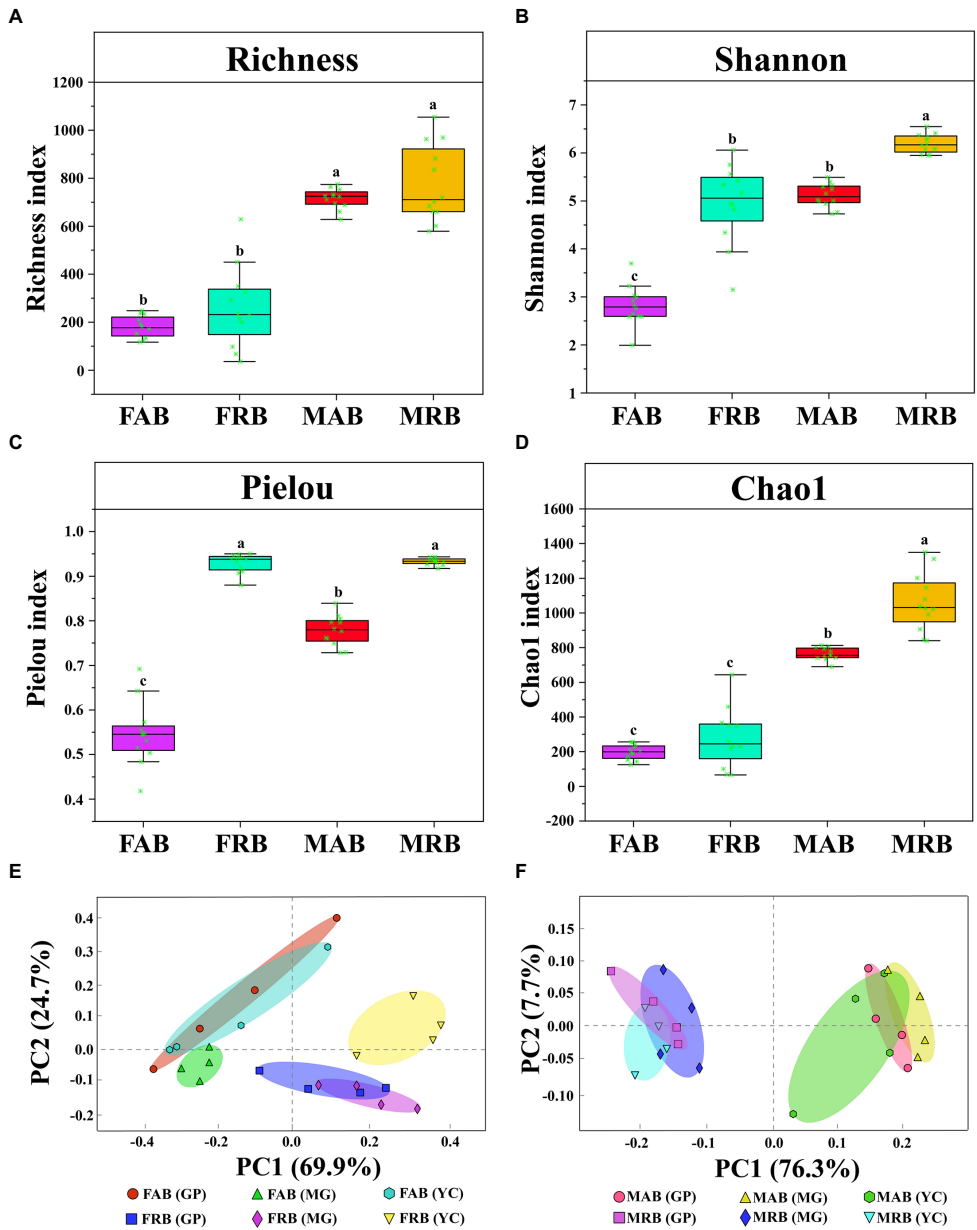
Alpha diversity and principal component analysis. **(A)** The Richness index of abundant and rare bacteria in the fruiting body and mycosphere. **(B)** The Shannon index of abundant and rare bacteria in the fruiting body and mycosphere. **(C)** The Pielou index of abundant and rare bacteria in the fruiting body and mycosphere. **(D)** The Chao1 index of abundant and rare bacteria in the fruiting body and mycosphere. **(E)** The principal component analysis of abundant and rare bacteria in the fruiting body. **(F)** The principal component analysis of abundant and rare bacteria in the mycosphere. Note: In Figures (A-D), there is no significant difference in Tukey’s test for the same letter on the boxplot (*p* > 0.05). Tukey’s test showed significant differences in patterns with different letters (*p* < 0.05). FAB, abundant bacteria of the fruiting body; FRB, rare bacteria of the fruiting body; MAB, abundant bacteria of the mycosphere; MRB, rare bacteria of the mycosphere; GP, Gaopo Township; MG, Mengguan Township; YC, Yangchang Town.

In addition, the diversity of abundant and rare taxa in different samples was analyzed. Alpha diversity index analysis showed that the diversity of MAB and MRB was significantly higher than that of fruiting bodies (*p* < 0.05) (Figures 4A–D). In both the fruiting body and mycosphere, the Shannon index of rare bacteria was substantially greater than that of abundant bacteria (*p* < 0.05) (Figure 4B). Additionally, the Pielou index of FRB was significantly higher than that of FAB (*p* < 0.05) (Figure 4C), and the Chao1 index of MRB was significantly higher than that of MAB (*p* < 0.05) (Figure 4D). Principal component analysis (PCoA) showed that abundant bacteria from different sites of the fruiting bodies and mycosphere clustered together, while rare bacteria also clustered together, and abundant and rare taxa were separated from each other (Figures 4E,F).

### 3.2. Analysis of community assembly, driving factors and niche breadth of the abundant and rare bacteria in the fruiting bodies and mycosphere

**Figure 5:**
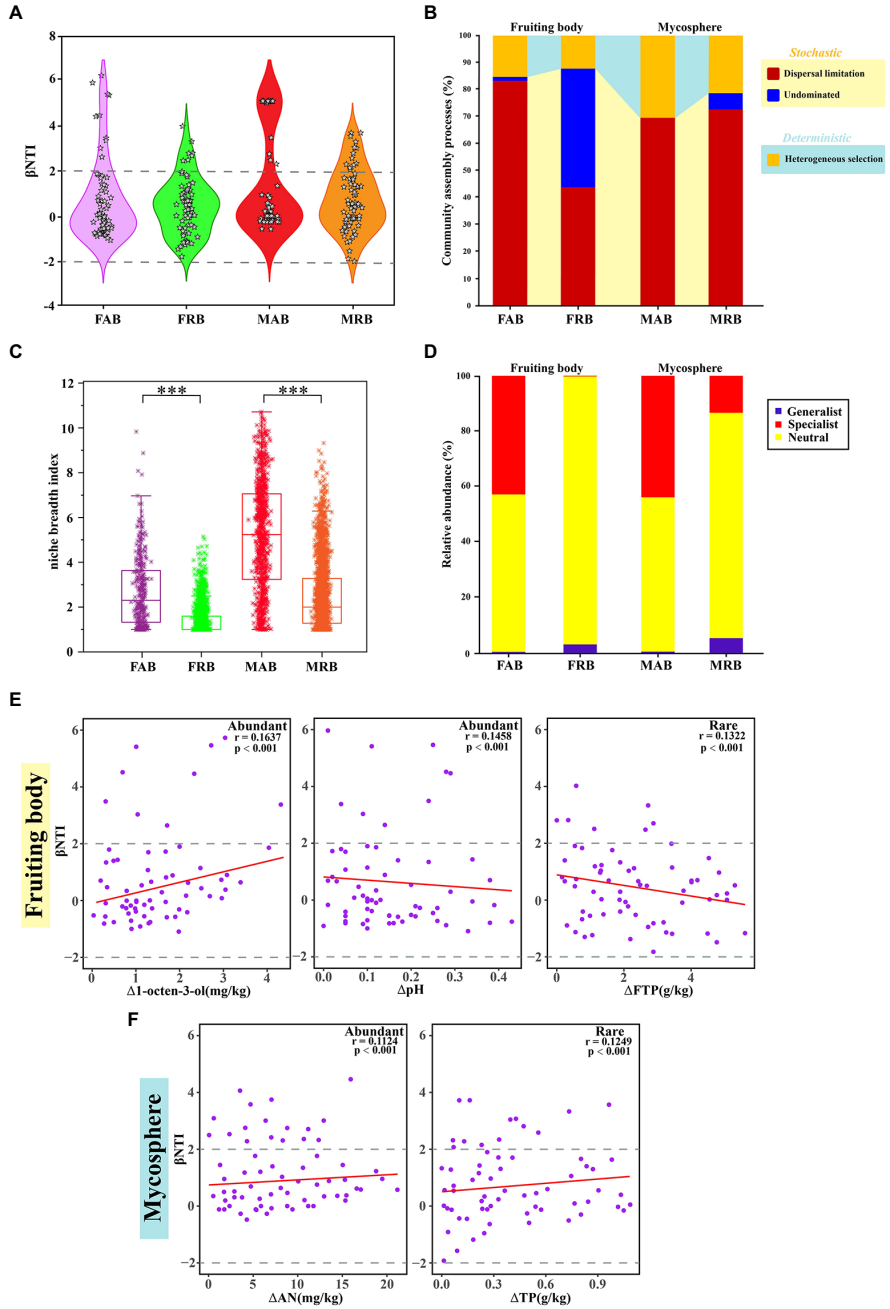
Null models and niche breadth index of the abundant and rare bacteria. **(A)** βNTI patterns of abundant and rare bacteria in the fruiting body and mycosphere. **(B)** The proportion of community assembly of the abundant and rare bacteria in the fruiting bodies and mycosphere is dominated by stochastic (dispersal limited and non-dominated) and partially deterministic (homogenous selection) processes. **(C)** Niche breadth of abundant and rare bacteria in fruitbody and mycosphere, these results were obtained based on the average of 100 bootstraps, and comparative analysis of abundant and rare bacteria within the sample was carried out using a *t*-test. * represent *p* < 0.05, ** represent 0.001 < *p* < 0.01, *** represent *p* < 0.001. **(D)** Relative abundance of the specialist, generalist, and neutral groups of abundant and rare bacteria in the fruiting bodies and mycosphere. **(E)** Spearman’s correlations between β-nearest taxon index (βNTI) of communities in the fruiting body and changes in environmental variables. **(F)** Spearman’s correlations between β-nearest taxon index (βNTI) of communities in the mycosphere soil and changes in environmental variables. FAB, abundant bacteria of the fruiting body; FRB, rare bacteria of the fruiting body; MAB, abundant bacteria of the mycosphere; MRB, rare bacteria of the mycosphere; FTP, total phosphorus of the fruiting body; AN, soil available nitrogen; TP, soil total phosphorus.

The null model shows the stochastic process for abundant and rare bacteria with a high relative contribution in the process of assembly (Figure 5A). For instance, in the stochastic process (dispersal limitation) of fruiting bodies, the relative contribution rate of the FAB (83.33%) was higher than that of the FRB (43.94%). In addition to being dominated by the dispersal limitation process, there were also some taxa in the “undominated” stochastic process in the fruiting bodies (Figure 5B). Similarly, the MAB and MRB were mainly restricted by the dispersal limitation of stochastic processes, but their proportion of deterministic processes (heterogeneous selection) was higher than that of the bacterial taxa in the fruiting bodies.

To explain the differences in the structure of abundant or rare bacterial communities in fruiting bodies and mycosphere of *C. cibarius*, the habitat specialization of the bacteria was examined. The niche breadth index of the OTU corresponding to the abundant and rare bacteria in the fruiting bodies and mycosphere, respectively, was estimated to further study the community structure. The niche breadth index of MAB was significantly higher than that of the FAB (Figure 5C). FAB (2.7 ± 0.084) had a significantly higher niche breadth index compared with that of the FRB (1.3801 ± 0.1527) (*p* < 0.001) (Figure 5C). MAB (5.19 ± 0.083) had a considerably larger niche breadth index compared with that that of MRB (2.54 ± 0.030) (*p* < 0.001) (Figure 5C). Furthermore, it is also important to distinguish between generalists and specialists to understand the assembly mechanisms of the microbial community. Therefore, we determined the generalist and specialist of abundant and rare bacteria in the two samples, respectively, depending on whether the niche breadth index was higher or lower than the simulated opportunity (Figure 5D). In the fruiting body and mycosphere, the proportion of specialists of abundant bacteria (FAB: 42.73%; MAB: 43.76%) was larger than that of rare bacteria (FRB: 0.05%; MRB: 13.23%), but the proportion of generalists of rare bacteria (FRB: 3.02%; MRB: 5.26%) was marginally higher than that of abundant bacteria (FAB: 0.29%; MAB: 0.52%) (Figure 5D).

To explore the major environmental factors that determined the assembly processes for abundant and rare taxa of the fruiting body and mycosphere, Mantel tests were conducted by comparing the βNTI values and each environmental variable (based on Euclidean distance matrices). The results suggested that many factors influenced the phylogenetic turnover of communities (Supplementary Table S3). The main explanatory variables were selected to determine the correlations between the assembly processes for different communities and environmental variables. The selected variables were 1-octen-3-ol and pH for FAB, FTP for FAB, AN for MAB, and TP for MRB (Supplementary Table S3). The significant positive correlation between 1-octen-3-ol and βNTI indicated that an increase in 1-octen-3-ol affected the assembly of the FAB (Figure 5E). However, the βNTI of FAB decreased with increasing pH (Figure 5E). Similar results were observed between βNTI and FTP in the FRB (Figure 5E). For the mycosphere, the βNTI of MAB decreased with the increase of AN, and the βNTI of MRB also showed a significant negative correlation with TP (Figure 5F).

### 3.3. Analysis of bacterial co-occurrence network between fruiting body and mycosphere

**Figure 6:**
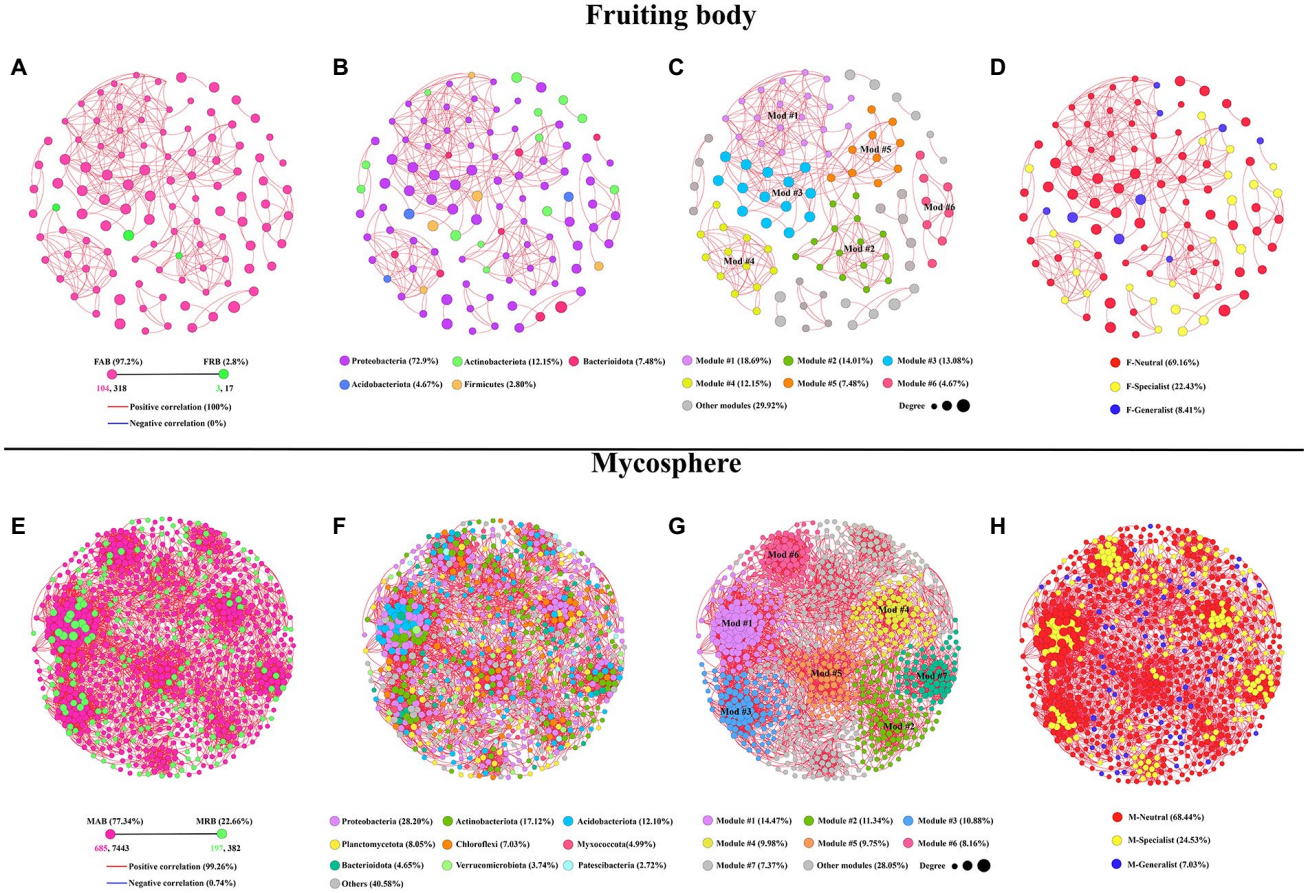
Analysis of the bacterial co-occurrence network between the fruiting body and mycosphere. **(A,E)** Depict the correlation co-occurrence network of abundant and rare bacteria in the fruiting body and mycosphere, respectively. **(B,F)** Depict the correlation co-occurrence network of bacterial taxonomic information in the fruiting body and mycosphere, respectively. **(C,G)** Depict the modularity of the bacterial correlation co-occurrence network of the fruiting body and microsphere, respectively. **(D,H)** Depict the type information of specialist, generalist, and neutral groups, respectively. Nodes of the network are colored according to abundant and rare bacteria information **(A,D)**, taxonomic information **(B,E)**, modularity **(C,F)**, and types of the generalist, specialist, and neutral group **(D,H)**. The size of the node depends on the degree of connectivity. Edge colors indicate positive (red) and negative (blue) correlations.

The co-occurrence networks of abundant and rare bacterial communities were further compared to assess their interactions. The results show that 107 nodes (OTUs) and 335 edges (correlations) were included in the samples of the fruiting body based on co-occurrence network analysis. Samples of the mycosphere contained 882 nodes and 7,825 edges. Other topological features were shown in Table 1. In the co-occurrence network, there were 318 edges and 104 nodes (97.2%) among the FAB, while the FRB had 3 nodes (2.8%) and 17 edges (Figure 6A). However, co-occurrence network of the MAB contained 7,443 edges and 685 nodes (77.34%). The overall number of MRB was 382 edges and 197 nodes (22.66%) (Figure 6E). Bacterial taxa in fruiting bodies and mycosphere are more likely to co-coexist (positive correlation, red line), rather than co-exclude (negative correlation, blue line). Negative correlations were 0 and 0.74% for the co-occurrence pattern of bacteria in the fruiting body and mycosphere, respectively, while positive correlations accounted for 100 and 99.26% in the co-occurrence network of the fruiting body and mycosphere (Figures 6A,E). Additionally, the predominant taxa in the fruiting body network were Proteobacteria (72.9%), Actinobacteriota (12.15%), and Bacteroidota (7.48%) (Figure 6B). Proteobacteria (28.20%), Actinobacteriota (17.12%), Acidobacteriota (12.10%), and Planctomycetota (8.05%) were the primary nodes in the network of the mycosphere (Figure 6F). The bacterial network in the fruiting body was clearly divided into six primary modules, according to the examination of network modularity data, and the proportion of OTUs in modules 1, 2, 3, and 4 was 18.69, 14.01, 13.08, and 12.15%, respectively (Figure 6C). However, the bacterial network in the mycosphere was divided into seven main modules, of which modules 1, 2, 3, and 4 accounted for 14.47, 11.34, 10.88, and 9.98% of the total OTUs, respectively (Figure 6G). Generalists, specialists, and neutral groups in the fruiting body and mycosphere were examined to assess the habitat specialization of the network. Most of the bacteria in both fruiting body and mycosphere samples belonged to neutral groups (F-neutral: 69.16%; M-neutral: 68.44%), and more specialists than generalists (F-generalist: 8.41%; M-generalist: 7.03%) were present (Figures 6D,H).

**Table 1:** Topological features of bacterial co-occurrence network in the fruiting body and mycosphere.

Treatments	OTUs number	Node	Edge	Modularity	AD	ACC	APD	GD
Fruiting body	118	107	335	0.691 ± 0.03	5.678	0.678	2.911	0.049
Mycosphere	944	882	7,825	0.791 ± 0.02	17.724	0.539	4.865	0.02

AD, average degree; ACC, average clustering coefficient; APD, average path distance; GD, graph density.

### 3.4. Correlation analysis of abundant and rare bacteria and environmental factors

**Figure 7:**
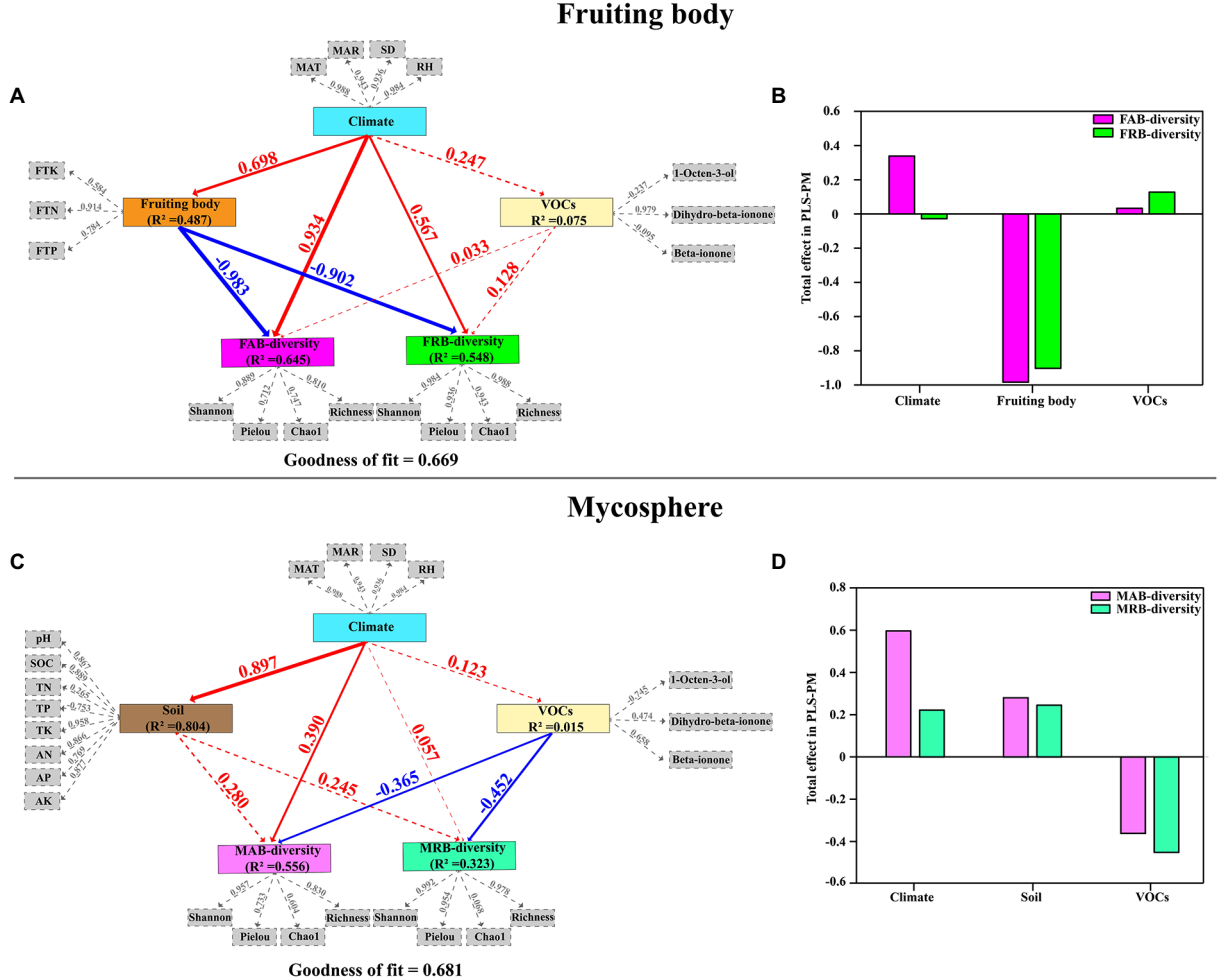
Partial least squares path modeling (PLS-PM) disentangling main pathways of climate, soil physicochemical properties, volatile organic compounds, and the chemical composition of the fruiting body on the diversity of abundant and rare bacteria in the fruiting body and mycosphere **(A,C)**; and the total effects of each variable on the diversity of abundant and rare bacteria in the fruiting body and mycosphere **(B,D)**. Each solid box represents a latent variable, and each gray dashed box represents an observed variable. The numbers on the arrow lines represent the normalized path coefficients, and the *R*^2^ values represent the variance of the dependent variable explained by the internal model. The width of the arrow reflects the magnitude of the path coefficient. The blue and red arrows represent positive and negative impacts, respectively. Solid and dashed lines indicate a significant (*p* < 0.05) and no significant (*p* > 0.05) correlation, respectively. The goodness of fit (Gof) statistic was used to test the model, and the Gof values of the fruiting body and microsphere soil were 0.669 and 0.681, respectively. MAT, mean annual temperature; MAR, mean annual rainfall; RH, relative humidity; SD, sunshine duration; FTN, total nitrogen of the fruiting body; FTP, total phosphorus of the fruiting body; FTK, total potassium of the fruiting body; pH, soil pH; SOC, soil organic matter; TN, soil total nitrogen; TP, soil total phosphorus; TK, soil total potassium; AN, soil available nitrogen; AP, soil available phosphorus; AK, soil available potassium; FAB-diversity, alpha diversity index of abundant bacteria in fruiting body; FRB-diversity, alpha diversity index of rare bacteria in fruiting body; MAB-diversity, alpha diversity index of abundant bacteria in mycosphere; MRB-diversity, alpha diversity index of rare bacteria in mycosphere.

Detailed exploration of the complex network of the relationship among climate, soil, VOCs, the chemical composition of the fruiting body, and the diversity of abundant and rare bacteria in the fruiting body and mycosphere was performed through PLS-PM of the fruiting body and mycosphere, respectively (Figure 7). The PLS-PM demonstrated a positive correlation between climate factors (MAT, MAR, SD, and RH) and the diversity of both FAB (0.934, *p* < 0.05) and FRB (0.567, *p* < 0.05) in the fruiting body. The diversity of both FAB (−0.983, *p* < 0.05) and FRB (−0.902, *p* < 0.05) was directly negatively impacted by the physicochemical properties of the fruiting bodies. However, the diversity of both FAB (0.033) and FRB (0.128) was positively impacted directly by VOCs (1-octen-3-ol, Dihydro-β-ionone, and β-ionone) (Figures 7A,B). Similarly, the climate factors were also positively correlated with the diversity of MAB (0.390, *p* < 0.05) and MRB (0.057, *p* < 0.05) in the mycosphere. The diversity of both MAB and MRB in the mycosphere was positively influenced by the soil physicochemical properties (pH, SOC, TN, TP, TK, AN, AP and AK) (0.280 and 0.245, respectively). However, VOCs exhibited adverse direct impacts on the diversity of MAB (−0.365, *p* < 0.05) and MAB (−0.452, *p* < 0.05) in the mycosphere (−0.365, *p* < 0.05; −0.452, *p* < 0.05) (Figures 7C,D).

### 3.5. Analysis of metabolic function, energy source, and symbiotic relationship of abundant and rare bacteria

**Figure 8:**
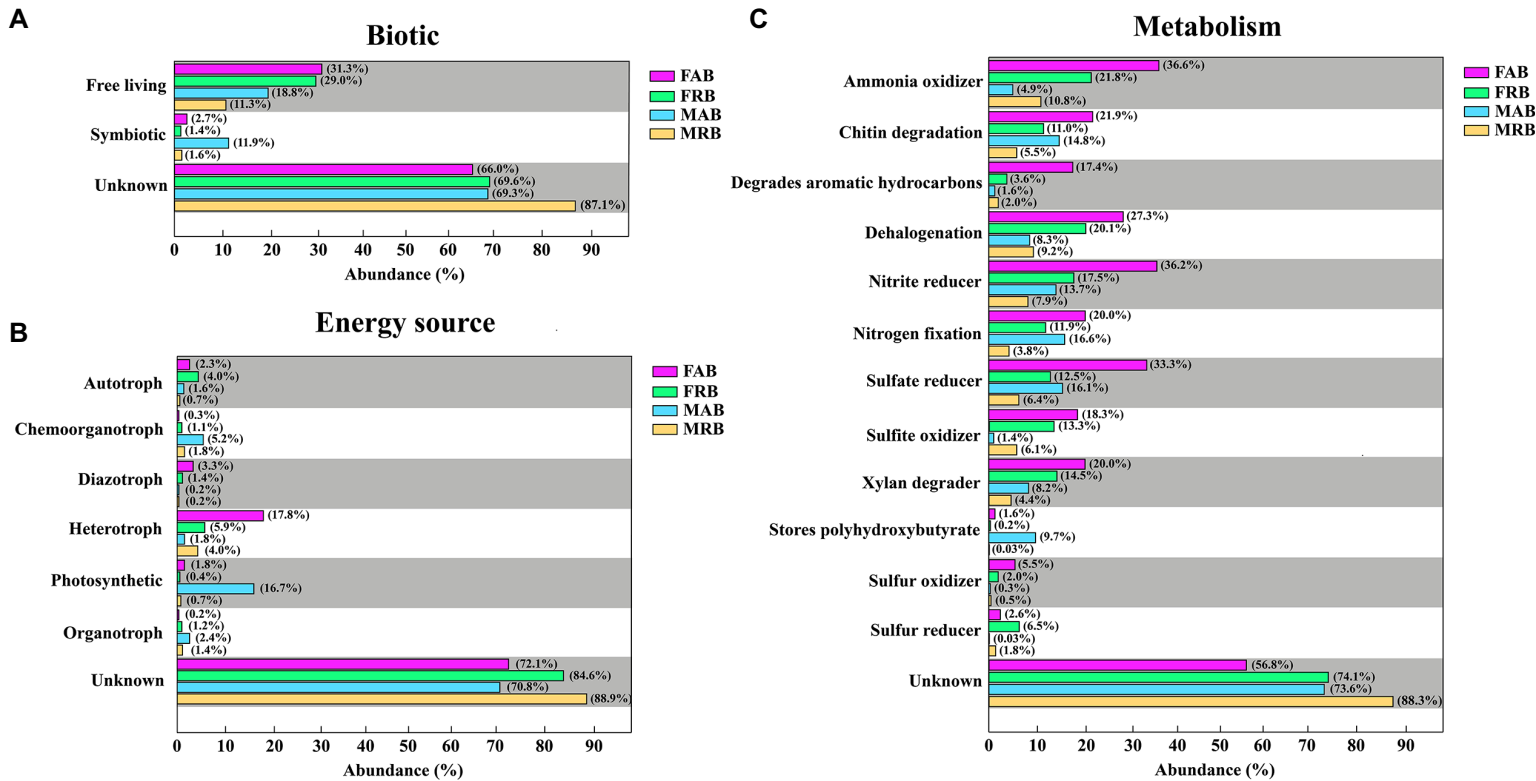
The potential phenotypic characteristics and metabolic functions of abundant and rare bacteria in the fruiting body and mycosphere. **(A)** Potential metabolism of abundant and rare bacteria in the fruiting bodies and mycosphere. **(B)** Energy sources of abundant and rare bacteria in the fruiting bodies and mycosphere. **(C)** Biological relationships between abundant and rare bacteria in the fruiting bodies and mycosphere. FAB, abundant bacteria of the fruiting bodies; FRB, rare bacteria of the fruiting bodies; MAB, abundant bacteria of the mycosphere; MRB, rare bacteria of the mycosphere.

From the analysis of potential biological relationships, most of the abundant and rare bacteria in the fruiting bodies and mycosphere were free-living, and the proportions of FAB (31.3%) and FRB (29.0%) in the fruiting bodies were higher than those in the mycosphere (MAB: 18.8%, MRB: 11.3%). In addition, the proportion of symbiosis in the MAB was relatively high (11.9%), which was higher than that in other taxa (Figure 8A). From the perspective of potential energy utilization, the FAB was heterotroph (17.8%), followed by diazotroph (3.3%) and autotroph (2.3%). The FRB was mainly heterotroph (5.9%) and autotroph (4.0%). However, the MAB was mainly photosynthetic (16.7%) and chemoorganotroph (5.2%). The MRB was mainly heterotroph (4.0%), and chemoorganotroph (1.8%) (Figure 8B). The results of potential metabolic functions showed that the FAB and FRB had similar functions, including ammonia oxidizer (FAB: 36.6%; FRB: 21.8%), nitrite reducer (FAB: 36.2%; FRB: 17.5%), sulfate reducer (FAB: 33.3%; FRB: 12.5%), dehalogenation (FAB: 27.3%; FRB: 20.1%) and chitin degradation (FAB: 21.9%; FRB: 11.0%). The MAB in the mycosphere were mainly nitrogen fixation (16.6%), sulfate reducers (16.1%), chitin degradation (14.8%), nitrite reducers (13.7%), and stores polyhydroxybutyrate (9.7%). However, the MRB in the mycosphere were ammonia oxidizer (10.8%), dehalogenation (9.2%), and nitrite reducer (7.9%) (Figure 8C).

## 4. Discussion

Most studies on fruiting bodies and mycosphere at the moment concentrate on the dominant genera of abundant bacterial taxa (Pent et al., 2017, 2020; Ge et al., 2021). In this study, we also examined the community structure, assembly, and symbiotic patterns of abundant and rare bacteria in the fruiting body and mycosphere, and we offered fresh insights into how environmental factors affect bacterial diversity in the habitat of *C. cibarius*.

### 4.1. Rare bacteria are indispensable members of the unique habitat of *Cantharellus cibarius*

The abundant taxa have traditionally received the majority of attention in soil microbial studies since they are typically regarded as the most active and significant participants in the biogeochemical cycle (Cottrell and Kirchman, 2003). However, recent research has emphasized the significance of rare taxa as hidden regulators of microbiome function (Lynch and Neufeld, 2015). Therefore, the abundant bacteria in the fruiting body and mycosphere only accounted for a small portion of the microbial diversity, according to both the proportion of the abundant and rare bacteria in OTUs (Figure 2) and the comparison of their diversity (Figure 4), which was consistent with the results of Pedrós-Alió (2012). This highlights that rare bacteria are the dominant force in community composition and diversity supplementation.

In addition, since the community composition of rare bacteria was significantly different from that of abundant bacteria, it should not be ignored in the microbial community composition. Proteobacteria were the most significant dominant group among the FAB and MAB, and the increase in their richness may be related to the higher nutrient status of the fruiting bodies in comparison with the mycosphere (Torsvik and Øvreås, 2002). The fruiting body and mycosphere of *C. cibarius* frequently contain Acidobacteriota as dominant taxa (Pent et al., 2017; Ge et al., 2021). Burke et al. (2006) described Acidobacteriota as mycorrhizal helper bacteria, which are close to the Proteobacteria group physiologically and ecologically, and have similar ecological niches in the rhizosphere/mycosphere (Singh et al., 2007; Kielak et al., 2016). Its effect on the growth and development of *C. cibarius* could not be ignored. In addition, Planctomycetota and Verrucomicrobiota which were two of the dominant phyla of rare bacteria in this study, are commonly found in the rhizosphere (Zul et al., 2007; da Rocha et al., 2009) and appear to have functionally strong rhizosphere capabilities, but their role in the growth and development of fruiting bodies remains to be demonstrated. Notably, Actinobacteriota and Firmicutes—two other dominant phyla of the rare bacteria in this study—were found to be associated with the production of VOCs in the study by Vahdatzadeh et al. (2015). Whether Actinobacteriota and Firmicutes are involved in the symbiosis of the unique aroma of *C. cibarius* deserves further study.

The dominant abundant bacteria of the fruiting bodies and mycosphere displays a trend toward functional type divergence (Ge et al., 2021). The findings of this study demonstrated that both abundant and rare bacteria had similar potential metabolic roles in the fruiting body and that the functional abundance of rare bacteria was not low. There is a difference in the functional distribution between the MAB and MRB in the mycosphere, which may be due to the diversity of rare bacteria increasing the functional redundancy of the mycosphere and providing complementary or unique metabolic pathways to support the function of the mycosphere ecosystem (Helliwell et al., 2013; Jousset et al., 2017). Potential energy utilization results revealed a high number of heterotrophs in both the FAB and FRB. This may be due to the exudates of the fruiting body and its underground hyphae (such as amino acids and trehalose), which serve as nutrient hotspots for numerous bacteria in soil and create diverse and unique ecological niches (Timonen et al., 1998; Rangel-Castro et al., 2002; Vellend, 2010). The abundant and rare bacteria in the fruiting body and mycosphere of *C. cibarius* were also discovered to be connected to the sulfur cycle in addition to the nitrogen cycle. This supports our first hypothesis and consistents with the findings of Liu et al. (2021) in the truffle, which suggest that bacteria of the fruiting bodies and mycosphere may have potential metabolic functions like sulfur cycling. Sulfur can be used by these bacteria as an energy source to support the development of fruiting bodies (Wang et al., 2019; Liu et al., 2021). From the analysis of potential biological relationships, most abundant and rare bacteria in the fruiting bodies and mycosphere of *C. cibarius* are free-living organisms, but there are some bacteria that are mainly in symbiotic, especially among the MAB in the mycosphere. The ability of fungal mycelia to actively migrate and grow in the soil may confer some specific resources that allow soil bacteria to respond positively to the growing mycosphere, select their preferred ecological niche, and form more symbiotic relationships (Noble et al., 2009; Warmink et al., 2009; Yun et al., 2013). Although fungal fruiting bodies and mycosphere structures may be transient (Haq et al., 2014), there is increasing evidence that bacteria play an important symbiotic function in specific fungal microflora (fruiting bodies and mycosphere) (Gohar et al., 2020; Yu et al., 2020; Ge et al., 2021). In summary, these findings reveal the importance of abundant and rare bacterial communities in the fruiting bodies and mycosphere of *C. cibarius*. In particular, the contributions of rare bacteria to community composition, diversity, and potential function cannot be ignored.

### 4.2. The assembly of abundant and rare bacterial communities in fruiting body and mycosphere is mainly a stochastic process but is also affected by different environmental factors

In this study, dispersal limitation was the main assembly process for the abundant and rare bacteria of the fruiting bodies and mycosphere of *C. cibarius*. Ma et al. (2017) and Xiao et al. (2018) also reported that soil bacteria were predominantly affected by dispersal limitation. Such stochastic processes allow species to coexist with overlapping niches and may regulate the diversity and ecosystem function of bacterial taxa in the fruiting body and mycosphere (Chase and Myers, 2011; Zhou and Ning, 2017). Additionally, some dormant bacteria can successfully serve as “seed banks” for bacterial sources when unfavorable circumstances restrict the growth of bacterial communities (Locey et al., 2020; Wisnoski et al., 2020). Thus, rare bacteria may contribute to the diversity of bacteria in the fruiting body and mycosphere of *C. cibarius*. However, compared with the mycosphere, the FAB and FRB in the fruiting body had a higher ratio of stochastic processes. This finding was consistent with a report by Chase (2014), which showed that the relative contribution of stochastic processes to the overall structure of soil microbial communities was increased over a small area. It is important to note that abundant bacteria were more affected by dispersal limitation in our current investigation compared with rare bacteria. This difference may be attributed to the different morphological characteristics of these bacterial taxa (an important integrated attribute of species related to their total abundance, growth rates, and range size) (Allen et al., 2006), and deterministic factors such as fruiting body habitat specificity (Hanson et al., 2012).

In the fruiting body and mycosphere samples, the proportion of specialists in the abundant bacteria was high (Figure 5D). This reflected that fruiting body and mycosphere habitat availability dominate specialists more than utilizing generalists with a wider range of habitat types, consistent with the findings of Munday et al. (1997) and Bean et al. (2002). It was recently reported that the symbiotic patterns of bacterial generalists differ in complexity and stability from those of specialists, which were more complex compared with those of generalists (Mo et al., 2020). In constast, we discovered that the proportion of generalists was higher in rare bacterial taxa, which were thought to have a wider niche breadth, stronger environmental tolerance, and more adaptable metabolic function (He Z. et al., 2022; He J. et al., 2022). These results indicated that rare bacteria played an active role in maintaining the functional diversification and stability of the ecosystem of the fruiting body and its microbiota. Neutral groups were still the main groups in the abundant and rare bacteria of the fruiting bodies and mycosphere. This could be explained by the fact that the homogenous environment supplied by fruiting bodies and the mycosphere reduced species niche differentiation and increased the proportion of neutral species in community aggregation (Bar-Massada et al., 2014).

It is essential to reveal the factors that lead to the dominance of different microbial community assembly processes in community ecology (Tripathi et al., 2018; Vass et al., 2020). Previous studies have revealed that soil pH and nutrient concentrations are the major determinants of the microbiome structure and functions in fungal fruiting bodies (Pent et al., 2017, 2018, 2020). However, this study found that the βNTI of FAB was negatively correlated with soil pH, while the βNTI of FRB was positively correlated with FTP. These findings indicated that the assembly processes of the FAB and FRB may be regulated by different environmental factors. In addition to soil pH and FTP, 1-octen-3-ol may be involved in the community assembly of FAB; however, the mechanism underlying the promotion of the FAB requires further investigation to prove a direct link. There was also a high positive correlation between the βNTI of MAB and the AN, while the MRB was positively correlated with TP. The influence of soil physicochemical properties on the bacterial community structure has been well explained (Rousk et al., 2010; Bahram et al., 2018). However, in addition to theoretical correlation analysis, it is necessary to verify the actual impact of each indicator through rigorous scientific design. Nevertheless, these results are consistent with our second hypothesis that different environmental factors influence the assembly process of abundant and rare bacterial communities in fruiting bodies and mycosphere of *C. cibarius*. To summarize, the assembly of abundant and rare bacterial communities in the fruiting bodies and mycosphere of *C. cibarius* was affected by different environmental factors, although the dispersal limitation in stochastic processes is dominant.

### 4.3. The complex and diverse symbiotic patterns of the bacteria in the mycosphere support development and formation of the fruiting body

The distribution and ecological function of microbes were also impacted by microbe–microbe interactions (Barberán et al., 2012). Network analysis and topological characteristics in this study revealed that from the mycosphere to the fruiting body, the correlation, complexity, and modularity of microorganisms gradually decreased. The complexity of the truffle microbial community was found to steadily decline from the bulk soil to the soil–truffle interface, and then to the fruiting body, which is consistent with the results of Liu et al. (2021). The following aspects could explain this phenomenon similar to “ecological filtering”: (1) the substrate released by fruiting bodies and their hyphae had an obvious influence on the resident bacteria, which was reflected in the marked difference of OTUs between fruiting bodies and the mycosphere; (2) the correlation was more significant in the mycosphere with higher microbial richness; (3) the bacterial community in the mycosphere had more functional modularity compared with that of the fruiting bodies, thus nutrient cycling and organic matter degradation were realized (Cong et al., 2015; Zhang et al., 2018b; Liu et al., 2021). This spatial heterogeneity may be crucial to understanding how different nutrient cycling processes at smaller spatial scales, including fruiting bodies and the mycosphere, are driven by more complex and diverse communities.

The bacterial co-occurrence network of the fruiting body and mycosphere of *C. cibarius* was consistently dominated by Proteobacteria and Actinobacteria, which may be more closely associated with metabolic processes such as nitrogen fixation and organic carbon consumption in the unique habitat of *C. cibarius* (Gohar et al., 2020; Ge et al., 2021). Additionally, the proportion of rare bacteria in the mycosphere was noticeably larger compared with that in the samples from fruiting bodies, which may be related to the intricacy of the mycosphere and the significance of rare bacteria in the global biogeochemical cycles (Hol et al., 2015; Jousset et al., 2017). In sum, the bacterial co-occurrence network of the mycosphere was more complex than that of the fruiting body, and this neework complexity may play an important role in the metabolism and nutrition needed for the growth and development of the fruiting body. In particular, the mechanism of the symbiosis of Proteobacteria still needs to be further evaluated.

### 4.4. The volatile organic compounds have different potential regulatory effects on bacterial diversity in the fruiting body and mycosphere

The diversity of abundant and rare bacteria in the fruiting body and mycosphere of *C. cibarius* in response to environmental conditions and host features were systematically evaluated by using PLS-PM. For both FAB and FRB in the fruiting bodies, their diversity was positively regulated by climate factors. Previous studies have shown that appropriate temperature, humidity, rainfall, and sunshine duration can increase soil microbial diversity and are also important conditions for the normal growth of soil fungal fruiting bodies (Xia et al., 2016; Wang et al., 2017; Sun et al., 2018; Yuichi, 2018). However, this study found that these appropriate climate factors also increase bacterial diversity in the fruiting body. This promoting effect may be a direct effect of climate factors, or it may be that climate factors stimulate *C. cibarius* to increase the production of soluble carbohydrates, thereby affecting the bacterial taxa depending on these compounds (Rangel-Castro et al., 2002). In contrast, the diversity of abundant and rare bacterial taxa was negatively regulated by the chemical composition of fruiting bodies. Previous studies have shown that the chemical composition of fungal fruiting bodies has a stronger influence on the diversity and community structure of specific bacteria in the fruiting bodies compared with the soil physicochemical properties (Pent et al., 2020). The ratios of N%, P%, C%, and C: N in fruiting bodies exhibit different degrees of influence on bacterial diversity (Pent et al., 2020). However, in this study, TN, TP, and TK of the fruiting body negatively regulated the diversity of both abundant and rare bacterial taxa. This may be because *C. cibarius* affect the composition and diversity of the microbial community near their fruiting bodies by changing soil conditions, thus forming small-scale and nutrient-rich hot spots (Pent et al., 2020). This would attract bacterial taxa with specific symbiotic functions to colonize the fruiting bodies and exclude other irrelevant bacteria, so as to effectively control the bacterial diversity. VOCs have a beneficial effect on the bacterial diversity of fruiting bodies. Relevant reports stated that the aromas of 1-octen-3-ol, dihydro-β-ionone, and β-ionone all had the potential to be attractive, having advantageous effects on luring animals and insects, and recruiting specific bacteria to colonize fruiting bodies (Thakeow et al., 2008; Sharma et al., 2012; Vahdatzadeh et al., 2015; Splivallo et al., 2019; Xu et al., 2019). Similar to EcMF, further research has been conducted on the interactions between VOCs and the microorganisms that are found in truffles, such as *Tuber melanosporium* and *Thelephora* (Antony-Babu et al., 2014; Vahdatzadeh et al., 2015; Benucci and Bonito, 2016; Splivallo et al., 2019). By generating VOCs, *Lyphyllum* attracted the colonization of *Burkholderia terrae* BS001(Haq et al., 2014). Thus, the fruiting body site of *C. cibarius* may be a significant aromatic dense niche that attracts particular bacteria. This is supported by bacteria with potential aromatic compound metabolic functions (Splivallo et al., 2015; Vahdatzadeh et al., 2015; Ge et al., 2021) and the PLS-PM results of this study (Figure 8), which show a close relationship between VOCs and diversity of the fruiting body and mycosphere. However, not all VOCs are synthesized by mycorrhizal fungi themselves, and endofungal bacteria in their fruiting bodies may also be involved in aroma formation (Splivallo and Ebeler, 2015; Saidi et al., 2016). In this process, bacteria can produce a variety of VOCs, and some VOCs have strong antibacterial activity (Ghasemi et al., 2021; Orban et al., 2023), which may have an opposite role on bacterial diversity of the fruiting body. For instance, Ghasemi et al. (2021) discovered that the VOCs produced by endofungal bacteria *Pseudomonas* sp. Bi1, *Bacillus* sp. De3, *Pantoea* sp. Ma3 and *Pseudomonas* sp. De1 isolated from wild growing mushrooms inhibited the activity of *Pseudomonas tolaasii* Pt18, the causal agent of mushroom brown blotch disease. Interestingly, the unique bacterial taxa in the fruiting body of *C. cibarius* can co-exist in the fruiting body of *C. cibarius* with many bacteria that produce antimicrobial metabolites such as antibiotics and VOCs. Further studies should be performed to determine whether it carries resistance genes or other symbiotic mechanisms.

The diversity of abundant and rare bacteria in the mycosphere of *C. cibarius* was also positively regulated by climate factors and soil physicochemical properties but had a significant negative correlation with VOCs (*p* < 0.05). This is consistent with our third hypothesis. Climate factors such as temperature, humidity, rainfall, and sunshine duration were reported as important factors affecting soil microbial diversity (Xia et al., 2016; Wang et al., 2017; Sun et al., 2018). Similarly, the positive regulation of soil physicochemical properties on bacteria in mycosphere has been documented in previous studies (Pent et al., 2017; Yu et al., 2020). However, it is more noteworthy that VOCs negatively regulate the diversity of abundant and rare bacteria in the mycosphere. Such results are similar to the recent hot phenomenon of “allelopathic inhibition.” Allelopathic inhibition, put simply, generally implies that the host species (plants, fungus, or microbes) will exhibit a variety of interaction processes and control species diversity when an organism introduces damaging macromolecules (allelopathic chemicals) into the environment (Rice, 1984). In relation to this, the compounds in this study—1-octen-3-ol and β-ionone—have been shown to exhibit antibacterial and antiviral properties (Beltran-Garcia et al., 1997; Zhu et al., 2010; Sharma et al., 2012; Kertesz and Thai, 2018). *Volvariella volvacea* was found to have the important fragrance component dihydro-ionone (Xu et al., 2019). Data from Vahdatzadeh et al. (2019) have further shown that there is a significant relationship between specific microbial classes and VOCs. Therefore, *C. cibarius* may release some VOCs to regulate the categories and diversity of bacteria in the mycosphere, so as to ensure the normal growth, development, and health of the fruiting body. Furthermore, it is claimed that *C. cibarius* have evolved a successful environmental strategy for acquiring and controlling the space they require to survive and reproduce (Warmink et al., 2009; Pent et al., 2017; Gohar et al., 2020; Pent et al., 2020; Ge et al., 2021). In this space, unique bacterial communities were assembled according to host identity, host chemical composition, physicochemical properties of the mycosphere, function guild, or other nutritional effects (Warmink et al., 2009; Pent et al., 2017; Gohar et al., 2020; Pent et al., 2020; Ge et al., 2021). Therefore, we speculated that VOCs of *C. cibarius* may provide another novel explanation for the competitiveness of *C. cibarius* and the formation of unique bacterial communities in the fruiting body and mycosphere, which will be further verified in the future studies. In summary, bacterial diversity in the fruiting bodies and mycosphere of *C. cibarius* is regulated by different environmental factors. Although VOCs are correlated with bacterial diversity in different ways, correlation does not always imply causation, and more data are needed to verify the specific interaction mechanism(s).

## Data availability statement

The datasets presented in this study can be found in online repositories. The names of the repository/repositories and accession number(s) can be found in the article/Supplementary material.

## Author contributions

YH, YZ, and GZ: conceptualization and funding acquisition. WG, YR, and CD: data acquisition. QS, YB, ZH, and TY: formal analysis. WG: writing the first draft. YH and SD: writing, review, and editing the manuscript. All authors have read and agreed to the published version of the manuscript.

## Funding

This work was financially supported by “Hundred” Talent Projects of Guizhou Province (Qian Ke He [2020] 6005), the Project of Guizhou Province Science and Technology (Qiankehe Platform for talents [2019] 5105), the Natural Science Foundation of China (Nos. 32160007, 32060011, 32260003), and Construction Program of Biology First-class Discipline in Guizhou (GNYL [2017] 009).

## Conflict of interest

The authors declare that the research was conducted in the absence of any commercial or financial relationships that could be construed as a potential conflict of interest.

## Publisher’s note

All claims expressed in this article are solely those of the authors and do not necessarily represent those of their affiliated organizations, or those of the publisher, the editors and the reviewers. Any product that may be evaluated in this article, or claim that may be made by its manufacturer, is not guaranteed or endorsed by the publisher.

## Footnotes

^1^ http://www.soortenbank.nl/

^2^https://github.com/OpenGene/fastp, version 0.20.0

^3^http://www.cbcb.umd.edu/software/flash, version 1.2.7

^4^ http://drive5.com/uparse/version7.1

^5^ http://data.cma.cn
