# Supplementary material for: New perspective: Symbiotic pattern and assembly mechanism of Cantharellus cibarius-associated bacteria
Source: Front Microbiol. 2023 Feb 16;14:1074468. doi: 10.3389/fmicb.2023.1074468 (PMC9978014; doi:10.3389/fmicb.2023.1074468)
Supplement: Supplementary file 1 [file Table_1.DOCX]

Supplementary Material

# Supplementary Tables

**Table S1.** Characteristics of mycosphere soil, fruiting body, climate and volatile organic compounds.

| **Site** | **pH** | **SOC**  **(g/kg)** | **TN**  **(g/kg)** | **TP**  **(g/kg)** | **TK**  **(mg/kg)** | **AN (mg/kg)** | **AP**  **(mg/kg)** | **AK**  **(mg/kg)** | **FTN**  **(g/kg)** | **FTP**  **(g/kg)** | **FTK**  **(g/kg)** | **MAT**  **(℃)** | **MAR**  **(mm)** | **SD**  **(h)** | **RH**  **(%)** | **1-octen-3-ol**  **(mg/kg)** | **Dihydro-β-ionone**  **(mg/kg)** | **β-Ionone**  **(mg/kg)** |
| --- | --- | --- | --- | --- | --- | --- | --- | --- | --- | --- | --- | --- | --- | --- | --- | --- | --- | --- |
| YC1 | 5.14 | 39.23 | 0.85 | 5.03 | 22.28 | 69.38 | 19.08 | 55.56 | 6.02 | 57.33 | 49.86 | 15.05 | 107.65 | 142.40 | 82.41 | 14.25 | 0.16 | 0.31 |
| YC2 | 5.15 | 47.95 | 0.89 | 5.24 | 21.08 | 68.20 | 20.91 | 61.02 | 5.86 | 58.16 | 48.92 | 15.05 | 107.65 | 142.40 | 82.41 | 11.22 | 0.17 | 0.33 |
| YC3 | 5.01 | 47.08 | 0.92 | 4.96 | 19.68 | 62.32 | 18.54 | 59.20 | 6.12 | 55.44 | 49.32 | 15.05 | 107.65 | 142.40 | 82.41 | 12.93 | 0.17 | 0.28 |
| YC4 | 4.97 | 40.97 | 0.90 | 5.19 | 19.88 | 64.02 | 19.42 | 56.17 | 6.32 | 56.02 | 47.25 | 15.05 | 107.65 | 142.40 | 82.41 | 12.54 | 0.18 | 0.29 |
| MG1 | 5.25 | 67.13 | 0.77 | 4.97 | 25.08 | 64.09 | 27.04 | 62.03 | 6.23 | 54.91 | 53.52 | 15.18 | 111.06 | 144.47 | 83.78 | 13.24 | 0.22 | 0.30 |
| MG2 | 5.23 | 55.21 | 0.78 | 5.20 | 26.48 | 67.62 | 34.06 | 61.82 | 6.52 | 55.45 | 55.39 | 15.18 | 111.06 | 144.47 | 83.78 | 11.56 | 0.20 | 0.31 |
| MG3 | 5.35 | 53.76 | 0.80 | 5.06 | 27.28 | 70.56 | 29.12 | 57.18 | 7.09 | 56.77 | 54.92 | 15.18 | 111.06 | 144.47 | 83.78 | 12.35 | 0.19 | 0.32 |
| MG4 | 5.26 | 51.73 | 0.82 | 4.63 | 25.88 | 74.67 | 32.38 | 58.39 | 6.75 | 55.68 | 54.32 | 15.18 | 111.06 | 144.47 | 83.78 | 10.21 | 0.17 | 0.35 |
| GP1 | 5.3 | 63.64 | 0.95 | 4.16 | 29.88 | 79.38 | 34.26 | 70.30 | 8.03 | 60.51 | 58.59 | 15.22 | 110.15 | 146.01 | 83.63 | 14.52 | 0.15 | 0.32 |
| GP2 | 5.4 | 75.85 | 0.86 | 4.70 | 30.68 | 76.44 | 29.17 | 74.95 | 7.32 | 60.21 | 57.92 | 15.22 | 110.15 | 146.01 | 83.63 | 12.05 | 0.17 | 0.29 |
| GP3 | 5.35 | 74.69 | 1.01 | 4.22 | 28.28 | 78.79 | 27.63 | 72.53 | 9.65 | 59.45 | 59.66 | 15.22 | 110.15 | 146.01 | 83.63 | 11.25 | 0.16 | 0.28 |
| GP4 | 5.25 | 74.10 | 0.91 | 4.96 | 29.68 | 83.49 | 28.77 | 68.29 | 6.53 | 58.32 | 58.76 | 15.22 | 110.15 | 146.01 | 83.63 | 13.25 | 0.19 | 0.27 |

MAT, mean annual temperature; MAR, mean annual rainfall; RH, relative humidity; SD, sunshine duration; FTN, total nitrogen of the fruiting body; FTP, total phosphorus of the fruiting body; FTK, total potassium of the fruiting body; pH, soil pH; SOC, soil organic matter; TN, soil total nitrogen; TP, soil total phosphorus; TK, soil total potassium; AN, soil available nitrogen; AP, soil available phosphorus; AK, soil available potassium.

**Table S2.** Alpha diversity index of abundant and rare bacteria in the fruiting body and mycosphere

| **Site** | **FAR** | **FRR** | **MAR** | **MRR** | **FAS** | **FRS** | **MAS** | **MRS** | **FAP** | **FRP** | **MAP** | **MRP** | **FAC** | **FRC** | **MAC** | **MRC** |
| --- | --- | --- | --- | --- | --- | --- | --- | --- | --- | --- | --- | --- | --- | --- | --- | --- |
| YC1 | 153 | 200 | 628 | 601 | 2.59 | 4.82 | 5.01 | 5.94 | 0.51 | 0.91 | 0.77 | 0.93 | 171.40 | 230.80 | 690.31 | 840.42 |
| YC2 | 172 | 234 | 661 | 662 | 2.59 | 4.94 | 4.72 | 6.07 | 0.50 | 0.90 | 0.72 | 0.94 | 205.00 | 259.16 | 752.12 | 844.78 |
| YC3 | 182 | 450 | 695 | 835 | 2.82 | 5.75 | 5.49 | 6.27 | 0.54 | 0.94 | 0.84 | 0.93 | 189.15 | 458.96 | 731.66 | 1079.12 |
| YC4 | 134 | 215 | 726 | 962 | 2.60 | 4.92 | 5.15 | 6.36 | 0.53 | 0.92 | 0.78 | 0.92 | 142.00 | 219.93 | 750.14 | 1201.58 |
| MG1 | 209 | 230 | 688 | 579 | 3.69 | 5.16 | 4.76 | 5.95 | 0.69 | 0.95 | 0.73 | 0.93 | 212.00 | 230.83 | 742.88 | 906.44 |
| MG2 | 248 | 629 | 711 | 702 | 3.02 | 6.05 | 4.98 | 6.18 | 0.54 | 0.94 | 0.76 | 0.94 | 256.57 | 643.25 | 741.50 | 1027.06 |
| MG3 | 152 | 98 | 733 | 684 | 3.22 | 4.34 | 4.94 | 6.15 | 0.64 | 0.94 | 0.75 | 0.94 | 152.25 | 99.00 | 793.22 | 1036.35 |
| MG4 | 192 | 350 | 723 | 719 | 2.91 | 5.56 | 5.24 | 6.08 | 0.55 | 0.95 | 0.79 | 0.92 | 193.66 | 352.04 | 758.54 | 1022.04 |
| GP1 | 123 | 36 | 774 | 969 | 2.75 | 3.15 | 5.39 | 6.41 | 0.57 | 0.87 | 0.81 | 0.93 | 223.43 | 66.00 | 807.00 | 1311.23 |
| GP2 | 234 | 325 | 753 | 1054 | 2.98 | 5.41 | 5.32 | 6.54 | 0.55 | 0.93 | 0.80 | 0.94 | 241.58 | 349.75 | 778.50 | 1349.12 |
| GP3 | 117 | 68 | 726 | 659 | 1.99 | 3.93 | 5.02 | 5.95 | 0.41 | 0.93 | 0.76 | 0.92 | 125.25 | 68.71 | 801.67 | 992.13 |
| GP4 | 237 | 293 | 764 | 882 | 2.64 | 5.33 | 5.28 | 6.33 | 0.48 | 0.94 | 0.79 | 0.93 | 254.65 | 366.37 | 812.75 | 1145.38 |

FAR, Richness index of abundant bacteria in fruiting body; FRR, Richness index of rare bacteria in fruiting body; MAR, Richness index of abundant bacteria in mycosphere; MRR, Richness index of rare bacteria in mycosphere; FAS, Shannon-Wiener index of abundant bacteria in fruiting body; FRS, Shannon-Wiener index of rare bacteria in fruiting body; MAS, Shannon-Wiener index of abundant bacteria in mycosphere; MRS, Shannon-Wiener index of rare bacteria in mycosphere; FAP, Pielou index of abundant bacteria in fruiting body; FRP, Pielou index of rare bacteria in fruiting body; MAP, Pielou index of abundant bacteria in mycosphere; MRP, Pielou index of rare bacteria in mycosphere; FAC, Chao1 index of abundant bacteria in fruiting body; FRC, Chao1 index of rare bacteria in fruiting body; MAC, Chao1 index of abundant bacteria in mycosphere; MRC, Chao1 index of rare bacteria in mycosphere.

**Table S3.** Mantel tests of environmental variables against the phylogenetic turnover (β-nearest taxon index) of abundant and rare taxa in the fruiting body and mycosphere.

| Variables | Fruiting body | | | | Mycosphere | | | |
| --- | --- | --- | --- | --- | --- | --- | --- | --- |
|  | Abundant-NTI | | Rare-NTI | | Abundant-NTI | | Rare-NTI | |
|  | r | p | r | p | r | p | r | p |
| pH | **0.3769** | **0.001** | 0.1086 | 0.134 | 0.0605 | 0.346 | -0.1230 | 0.757 |
| SOC | -0.1193 | 0.822 | -0.1320 | 0.863 | 0.0903 | 0.224 | -0.0458 | 0.587 |
| TN | -0.0220 | 0.532 | 0.0268 | 0.394 | 0.1017 | 0.119 | 0.0285 | 0.374 |
| TP | -0.1597 | 0.872 | 0.0743 | 0.316 | 0.0854 | 0.191 | **0.2182** | **0.017** |
| TK | -0.1558 | 0.915 | -0.0864 | 0.724 | 0.1675 | 0.079 | -0.0328 | 0.515 |
| AN | -0.0906 | 0.746 | -0.0556 | 0.588 | **0.1917** | **0.024** | -0.0322 | 0.507 |
| AP | -0.0422 | 0.609 | -0.0159 | 0.506 | 0.1089 | 0.145 | 0.1736 | 0.067 |
| AK | 0.1092 | 0.149 | -0.0984 | 0.744 | -0.1356 | 0.826 | 0.0971 | 0.212 |
| FTN | 0.1021 | 0.146 | 0.0294 | 0.346 | -0.1035 | 0.6 | 0.0663 | 0.25 |
| FTP | -0.0538 | 0.621 | **0.1772** | **0.043** | -0.1461 | 0.892 | 0.0985 | 0.21 |
| FTK | -0.1854 | 0.971 | -0.0292 | 0.525 | 0.1355 | 0.087 | -0.0119 | 0.442 |
| MAT | -0.1339 | 0.893 | -0.1129 | 0.834 | 0.0899 | 0.122 | -0.057 | 0.656 |
| MAR | 0.03238 | 0.336 | 0.0785 | 0.263 | 0.1202 | 0.094 | -0.1161 | 0.841 |
| SD | -0.1663 | 0.925 | -0.1166 | 0.874 | 0.1572 | 0.079 | -0.0246 | 0.483 |
| RH | 0.1426 | 0.071 | -0.0967 | 0.748 | 0.1747 | 0.052 | -0.0963 | 0.746 |
| 1-octen-3-ol | **0.1826** | **0.026** | -0.2147 | 0.954 | -0.0921 | 0.64 | 0.0619 | 0.309 |
| Dihydro-β-ionone | 0.1145 | 0.083 | -0.0576 | 0.569 | -0.0364 | 0.5 | 0.1343 | 0.085 |
| β-Ionone | 0.1286 | 0.118 | -0.1618 | 0.852 | 0.0676 | 0.323 | -0.1445 | 0.854 |

MAT, mean annual temperature; MAR, mean annual rainfall; RH, relative humidity; SD, sunshine duration; FTN, total nitrogen of the fruiting body; FTP, total phosphorus of the fruiting body; FTK, total potassium of the fruiting body; pH, soil pH; SOC, soil organic matter; TN, soil total nitrogen; TP, soil total phosphorus; TK, soil total potassium; AN, soil available nitrogen; AP, soil available phosphorus; AK, soil available potassium.
